# Profiling of Anti‐FVIII Antibodies in Acquired Haemophilia A: ‘Insights into Domain Specificity, Isotype Variability, and Clinical Correlations’

**DOI:** 10.1111/hae.70056

**Published:** 2025-05-05

**Authors:** Ann‐Cristin Berkemeier, Isabell Matuschek, Katrin Hartlieb, Thilo Albert, Natascha Marquardt, Johannes Oldenburg, Behnaz Pezeshkpoor

**Affiliations:** ^1^ Institute of Experimental Hematology and Transfusion Medicine Medical Faculty, University Hospital Bonn, University of Bonn Bonn Germany; ^2^ Center for Rare Diseases Bonn (ZSEB) University Clinic Bonn Bonn Germany

**Keywords:** acquired haemophilia A, anti‐FVIII antibodies, bleeding, FVIII inhibitors, immunoglobulin isotypes, mortality

## Abstract

**Introduction:**

Acquired haemophilia A (AHA) is a rare autoimmune disorder caused by autoantibodies against coagulation factor VIII (FVIII), resulting in significant bleeding risks.

**Aim:**

To characterize the anti‐FVIII antibody profile in AHA patients by assessing isotypes, subclasses, and correlations with key clinical parameters.

**Methods:**

Eighty AHA patients were retrospectively analysed by assessing FVIII inhibitor levels, antibody isotypes (IgG, IgA, IgM), IgG subclasses, and domain specificity using a bead‐based assay. Clinical data were correlated with antibody profiles. IgG domain profiles were compared with a congenital haemophilia A (CHA) cohort.

**Results:**

The cohort had a median age of 74 years, with 60% males. Idiopathic cases accounted for 67%, and 17% had bleeding linked to medical interventions. Major bleeding sites were musculoskeletal/retroperitoneal (45%) and skin (36%). Within six months, 18% of patients died, mostly from sepsis. Anti‐FVIII IgG antibodies were present in all patients, with IgG_4_ (96%) and IgG_3_ (60%) being the most common subclasses. IgM and IgA anti‐FVIII antibodies were detected in 17.5% and 18.8% of patients, respectively, with IgM positivity associated with higher mortality (33%). IgG_4_ subclass correlated significantly with inhibitor titres (*r*_s_ = 0.54; *p* < 0.001). Compared to CHA, AHA showed a higher prevalence of C1C2 domain‐targeting antibodies (49% vs. 77%), associated with NBA levels (*r*_s_ = 0.51; *p* < 0.001).

**Conclusion:**

Anti‐FVIII antibody profiling reveals distinct patterns in AHA, with IgG_4_ linked to higher inhibitor levels. The C1C2 domain specificity of the anti‐FVIII antibodies suggests a potential role of this FVIII domain in the immunopathology of AHA patients, warranting further investigation to improve prognostic tools.

## 1 Introduction

Acquired haemophilia A (AHA) is a rare and life‐threatening autoimmune disorder characterized by the development of autoantibodies against coagulation factor VIII (FVIII). This condition disrupts normal haemostasis, leading to spontaneous bleeding or excessive haemorrhage [1]. With a low incidence of 1–1.5 per million per year, AHA predominantly affects older adults and poses significant challenges in diagnosis and management, with high morbidity and mortality rates if not properly treated [2]. The patients' laboratory findings show a prolonged activated thromboplastin time (aPTT) and reduced FVIII activity (FVIII:C) [3]. To confirm the suspected diagnosis, the Nijmegen‐Bethesda assay (NBA) is used to detect inhibitory anti‐FVIII antibodies [4].

It has been proposed that immunological characterization of autoantibodies by enzyme‐linked immunosorbent assays (ELISA) could serve as a complementary tool in diagnosing AHA and may offer prognostic value. To assess correlations between antibody profiles and clinical outcomes, ELISA‐based assays have been utilized in studies by the Quebec Reference Center for Inhibitors (QCRI) and the GTH‐AH 01/2010 study [5, 6]. These studies focused on profiling immunoglobulins and identifying differences in the affinity of anti‐FVIII subclasses in AHA patients. Notably, certain immunoglobulin isotypes, particularly in the GTH study, were associated with poorer outcomes and increased mortality, underscoring the potential of antibody profiling in risk stratification for AHA.

Although numerous studies in congenital haemophilia A (CHA) patients have investigated the domain specificity of anti‐FVIII antibodies, knowledge on this topic remains limited in AHA [7, 8]. Some investigations have used FVIII domains or multi‐domain structures produced in cell culture or bacterial strains to detect domain‐specific or multi‐domain‐specific anti‐FVIII antibodies via immunoblotting [9] or ELISA [10]. This knowledge gap underscores the need for further research into domain‐specific antibody profiles in AHA, which could potentially reveal prognostic markers for this unique patient group.

Previous studies indicate that AHA autoantibodies show considerable variability in both isotype and binding domain, factors that can influence disease severity and therapeutic response [5, 11]. For instance, distinct antibody isotypes or subclasses may correlate with higher inhibitor levels, while specific domain targets (e.g., C2 or A2) are thought to interfere with FVIII function in ways that can exacerbate bleeding severity. The functional domains A2, A3, and C2 are of particular interest, as antibodies binding to these regions can disrupt FVIII's interactions with factor X and factor IXa (A2, A3) or with phospholipids and von Willebrand factor (C2) [12]. However, comprehensive data linking anti‐FVIII antibody profiles to clinical outcomes such as bleeding severity, transfusion requirements, and mortality remain sparse. This lack of data limits our ability to stratify AHA patients based on risk and tailor therapies accordingly.

In this study, we aimed to characterize a cohort of 80 AHA patients by investigating the relationship between antibody profiles and key clinical parameters, including FVIII activity, haemoglobin levels, and inhibitor titres. Additionally, we explored the distribution of IgG subclasses (IgG_1‐4_), isotype overlap (IgG, IgM, IgA), and their domain specificity to assess their potential impact on clinical outcomes.

## 2 Material and Methods

### 2.1 Patients

Eighty AHA patients, treated at our centre between 2010 and 2022, were included in this study. Plasma samples from diagnosis were retrospectively analysed using Luminex immunoassay. Clinical data, including demographics, underlying disorders, bleeding causes and sites, FVIII activity, NBA levels, and mortality outcomes, were collected from patient records (Table 1). Over the 12‐year data collection period, treatment strategies for AHA evolved considerably, reflecting a shift toward more individualized care. This included various immunosuppressive therapies (e.g., corticosteroids, cytotoxic agents, and intravenous immunoglobulins), immunapheresis, and the introduction of targeted antibody treatments. Consequently, treatment decisions for the 80 patients were individualized based on factors such as pre‐existing conditions, underlying causes of AHA, and patient‐specific therapeutic goals. Additionally, a comparative cohort of 41 CHA patients with inhibitors, was analysed. The characteristics of this cohort are shown in Table S1. The study was approved by the Bonn University Medical Sciences ethics committee (approval 183/07), and all patients provided written consent in accordance with the Declaration of Helsinki.

**TABLE 1:** Patient cohort characteristics.

Age (years)	Median	74
	Range	25–90
Gender [*n* (%)]	Male	50 (60)
	Female	30 (40)
Underlying disorder [*n* (%)]	Idiopathic	54 (67)
	Solid neoplasia	11 (14)
	Auto‐immune‐diseases	7 (9)
	Haematologic neoplasia	4 (5)
	No data	4 (5)
	Pregnancy	0
	Drug‐induced	0
Cause of bleeding [*n* (%)]	Spontaneous	58 (73)
	Medical intervention	14 (17)
	(Surgery, endoscopic, biopsy, dentist, injections, access)	
	No data	6 (7)
	Trauma	2 (3)
Location of bleeding [*n* (%)]	Musculoskeletal	30 (38)
	Skin	29 (36)
	Mucosa	20 (25)
	No data	6 (8)
	Retroperitoneal	5 (6)
	Chatheter devices (artery access, central venous catheter)	4 (5)
	Hemarthrosis	2 (3)
	Central nervous system	1 (1)
Mortality [*n* (%)]	Deceased	14 (18)
	Survival	62 (77)
	No data	4 (5)
Haemoglobin [g/dL]	Median	8.7
	Range	4.2–14.7
FVIII activity (IU/dL)	Median	< 1
**Baseline FVIII inhibitor concentration (BU/mL)**		
NBA (Assay independent) (*n* = 80)	Median	19
	Range	1.4–791

NBA (chromogenic FVIII assay) (*n* = 36)	Median	16
	Range	1.7–791

NBA (one stage FVIII assay) (*n* = 44)	Median	24
	Range	1.4–549

NBA titre subgroups (*n* = 80) [*n* (%)]	< 5 BU/mL	17 (21)
	≥ 5 BU/mL	63 (79)
Inhibitor kinetic [*n* (%)]	Type I (linear)	41 (51)
	Type II (non‐linear)	39 (49)

Abbreviations: g/dL: gram/deciliter; IU/dL: International Units/deciliter; *n* = number; NBA: Nijmegen‐Bethesda assay.

### 2.2 NBA

NBA was performed as previously described with modifications [3, 4]. Briefly, heat‐inactivated plasma samples were serially diluted with imidazole‐BSA buffer (IB), and imidazole‐buffered control plasma (pFVIII) was added (1+1) as a source of FVIII. After incubation of the mixtures at 37°C for 2 h, FVIII activity (FVIII:C) was measured on an Atellica Coag 360 analyzer (Siemens Healthineers, Munich, Germany), using the Siemens one‐stage clotting assay using APTT‐SP (Werfen, Barcelona, Spain, REF: 20006300) using Helena FVIII deficient plasma (Helena Biosciences, Gateshead, UK, REF: HE5193) (*n* = 44) or a chromogenic FVIII assay based on bovine factors (REF: B4238‐40, Siemens Healthineers) (*n* = 36). Inhibitor titres were calculated as Bethesda Units (BU/mL), wherein one BU is defined as the amount of inhibitor in the patient's plasma, which inactivates 50% of FVIII:C. A positive result of the NBA was defined as ≥ 0.6 BU/mL.

### 2.3 Luminex Assay for Identification of Anti‐FVIII Antibodies

For anti‐FVIII antibody measurement, MagPlex Microspheres (Luminex DiaSorin, Saluggia, Italy) were coupled covalently by a 2‐step carbodiimide reaction as previously described [13] using the xMAP Antibody Coupling Kit (Luminex) following the manufacturer's instructions. The Luminex epitope Mapping (LumiTope) assay was performed as previously described [8, 13]. To establish the mean fluorescence intensity (MFI) cut‐off for positivity of each bead, a multistep standardization protocol was used as previously described [8, 13]. A run‐specific floating cut point was established for each bead by testing a negative control (NC) following calculation according to the formula: Floating cut point = [Mean log‐transformed MFI of NC pool] × [CF]. Similar to other studies and due to the overall low MFI values of the NC for the IgG subclasses the cut‐off was calculated as cut point = [Mean log‐transformed MFI of NC pool] × [CF] × 2 [14, 15]. Due to the overall low MFI values of the NC for the IgA, cut‐off was calculated as cut point = [Mean log‐transformed MFI of NC pool] × [CF] × 3.

A sample was assigned as LumiTope positive, if the MFI signal of at least one of the two FVIII‐coupled beads (FL_FVIII_ or BDD_FVIII_), exceeded the cut‐off. To confirm a positive result, patient samples were incubated with 200 IU of Octocog alfa (Bayer, Leverkusen, Germany) for 15 min at 37°C. The result was regarded as specific if there was a 20% or greater reduction in MFI signal following FVIII spiking.

### 2.4 Statistical Analysis

Statistical analysis and figures were conducted using Graph pad prism 10.2.2 software (Boston, USA), respectively. Patient data correlations were calculated using nonparametric Spearman's rank‐order correlation test (*r*_s_) and correlation analysis was calculated using Pearson correlation coefficient. The correlation is considered not significant if the *p* value ≥ 0.05. The *p* value of the mortality rate was calculated by Fisher's exact test. The tables were created using Microsoft Exel Version 2021 (Microsoft Corporation, Redmond, USA). Venn diagrams were designed using Creately software (Melbourne, Australia).

## 3 Results

### 3.1 Patient Cohort Characteristics

The study cohort comprised 80 patients with AHA. Of these, 63 patients had high‐titre inhibitors (> 5 BU/mL), while 17 had low‐titre inhibitors (< 5 BU/mL). The overall median NBA level was 19 BU/mL, with a median of 24 BU/mL for clotting‐based assay and 16 BU/mL for chromogenic assay. The median age of the cohort was 74 years with no pregnancy‐related cases, but there was one young patient who developed AHA as part of an autoimmune aetiology (Table 1).

For 67% of the patients, the cause of AHA was idiopathic. In 17% of cases, bleeding was associated with a medical intervention, while 73% were spontaneous. The most common bleeding sites were musculoskeletal or retroperitoneal areas (45%), followed by the skin (36%). Fourteen patients (14/76; 18%, 4 patients no data) died within 6 months of diagnosis, with sepsis being the leading cause of death. Due to variations in transfusion practices across departments, haemoglobin levels, rather than transfusion numbers, were used to assess the severity of bleeding. The median haemoglobin level in the cohort was 8.7 g/dL, ranging from 4.2 to 14.7 g/dL.

### 3.2 Anti‐FVIII Antibody Isotype and Subclass Distribution in AHA Patients

**FIGURE 1:**
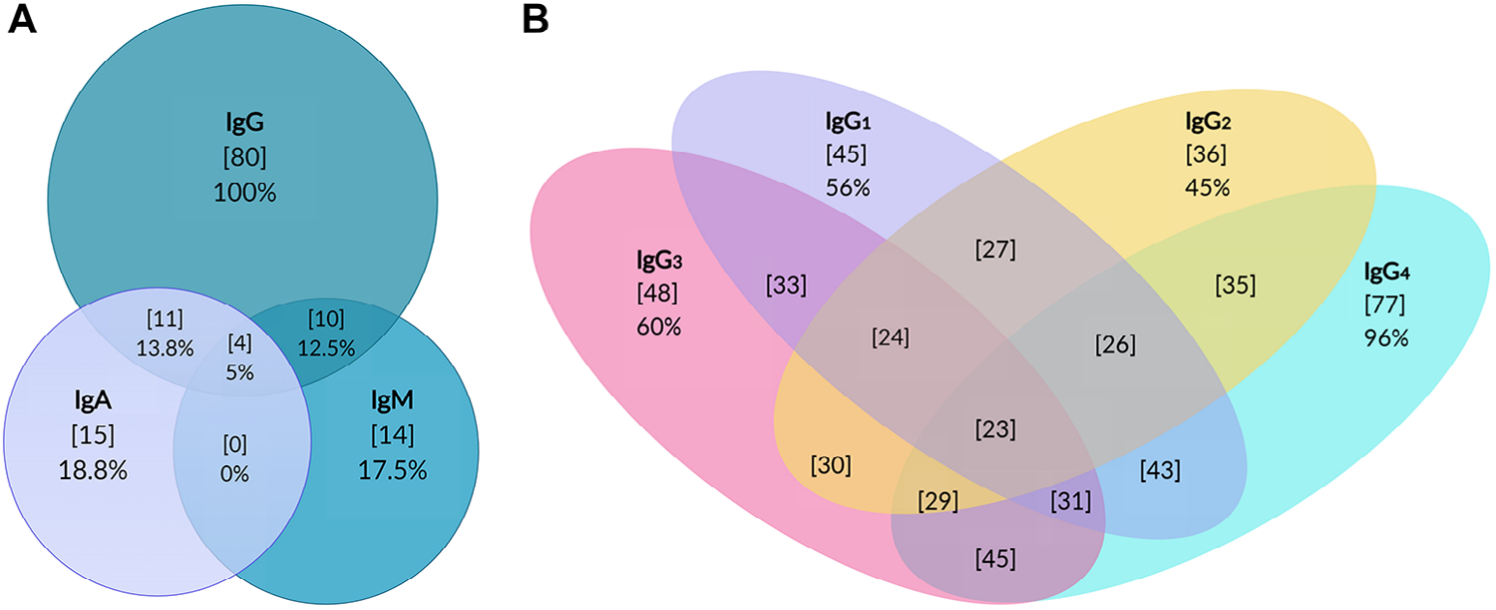
Venn diagram showing patients positive for any combination of immunoglobulin isotype and subclass of anti‐FVIII antibodies. (A) Number of positive patients in the corresponding Ig classes [*n*], % positive (out of 80 patients). The overlaps of the circles correspond to patients with more than one isotype. (B) Number of positive patients in the corresponding IgG subclass [*n*], % positive (out of 80 patients). The overlaps of the circles correspond to patients with more than one subclass. IgG, IgG_1_, IgG_2_, IgG_3_, IgG_4_, IgM, IgA = Immunoglobulin G, ‐G_1_, ‐G_2_, ‐G_3_, ‐G_4_, M and A.

All AHA patients tested positive for anti‐FVIII IgG antibodies. Additionally, 15 patients (18.8%) tested positive for IgA antibodies, and 14 patients (17.5%) were positive for IgM antibodies. Notably, 4 patients (5%) were tested positive for all three isotypes (Figure 1A, Venn Diagram). Subclass analysis of IgG revealed that 45 patients (56%) were positive for IgG_1_, 36 patients (45%) for IgG_2_, 48 patients (60%) for IgG_3_, and 77 patients (96%) for IgG_4_ antibodies. The most common antibody combination is between IgG3 and IgG4 (45/80 patients; 56%) (Figure 1B, Venn Diagram).

### 3.3 Correlation of Anti‐FVIII Antibodies with Clinical Outcomes, FVIII:C, NBA and Haemoglobin Levels

When correlating the anti‐FVIII antibody profile with the clinical outcome of the patients, the analysis revealed that IgM positive patients (4 deaths/12 patients, 2 patients unknown) had a higher mortality rate (33%) than the IgM negative patients (16%). While the Fisher's exact test result of *p* = 0.22 for mortality analysis indicates no significant finding in the current dataset, increasing the sample size in future studies could provide more clarity. Nevertheless, it is an indication of a possible connection. On the other hand, IgA positive patients had a similar mortality rate compared to IgA negative patients (14% vs. 19%) (Table 2). No significant correlation was identified between the anti‐FVIII antibody isotype patterns and clinical parameters such as the underlying cause of the disease or the location or type of bleeding. Specifically, the presence or distribution of antibody isotypes (IgG, IgA, or IgM) did not show any association with disease aetiology, whether idiopathic or related to medical intervention. Similarly, the site of bleeding—whether musculoskeletal, skin, or other areas—did not correlate with the antibody profiles.

**TABLE 2:** Isotype specific mortality rate of anti‐FVIII antibodies.

	Patients	Death	Survival	No data	Mortality in % [calculation]
IgG	Positive (80)	14	62	4	18% [14/76]
	Negative (0)	na	na	na	na
IgM	Positive (14)	4	8	2	33% [4/12]
	Negative (66)	10	54	2	16% [10/64]
IgA	Positive (15)	2	12	1	14% [2/14]
	Negative (65)	12	50	3	19% [12/62]

*Note*: Mortality rate was calculated by differentiating between Isotype positive and negative patients. For some patients, survival or death data at 6 months were not available in our records (no data).

Abbreviations: IgG, IgM, IgA = Immunoglobulin G, M and A; na = not applicable.

Overall, anti‐FVIII IgG antibodies demonstrated a strong correlation to NBA levels with both FL_FVIII_ (*r*_s_ = 0.37; *p* < 0.001) and BDD_FVIII_ beads (*r*_s_ = 0.57; *p* < 0.001). Notably, IgG_2_ antibodies showed a significant correlation with BDD_FVIII_ (*r*_s_ = 0.31; *p* = 0.01), whereas IgG_4_ antibodies correlated to inhibitor titres with both FL_FVIII_ (*r*_s_ = 0.45; *p* < 0.001) and BDD_FVIII_ (*r*_s_ = 0.54; *p* < 0.001) beads (Table 3 and Figure S1).

**TABLE 3:** Correlation of anti‐FVIII Isotype and FVIII‐activity, NBA, haemoglobin.

			Correlation with FVIII activity		Correlation with NBA		Correlation with haemoglobin levels	
Ig specificity	Positive patients (*n*)	Bead region	*r*_s_	*p* value	*r*_s_	*p* value	*r*_s_	*p* value
IgG	80	FL_FVIII_	**−0.45**	**0.00**	**0.37**	**0.00**	**−0.28**	**0.02**
		BDD_FVIII_	**−0.36**	**0.00**	**0.57**	**0.00**	**−0.26**	**0.03**
IgG_1_	45	FL_FVIII_	−0.17	0.14	0.00	0.98	**−0.28**	**0.02**
		BDD_FVIII_	−0.09	0.44	0.16	0.18	**−0.26**	**0.03**
IgG_2_	36	FL_FVIII_	−0.20	0.08	0.11	0.35	**−0.39**	**0.00**
		BDD_FVIII_	−0.21	0.07	**0.31**	**0.01**	**−0.37**	**0.00**
IgG_3_	48	FL_FVIII_	**−0.32**	**0.00**	0.09	0.48	**−0.30**	**0.01**
		BDD_FVIII_	−0.20	0.07	0.11	0.35	**−0.31**	**0.01**
IgG_4_	77	FL_FVIII_	**−0.32**	**0.00**	**0.45**	**0.00**	−0.23	0.06
		BDD_FVIII_	−0.21	0.07	**0.54**	**0.00**	−0.21	0.08
IgM	14	FL_FVIII_	0.21	0.47	−0.44	0.13	−0.19	0.60
		BDD_FVIII_	0.01	0.97	−0.12	0.70	0.35	0.32
IgA	15	FL_FVIII_	−0.12	0.67	−0.12	0.76	−0.04	0.90
		BDD_FVIII_	0.04	0.88	−0.48	0.17	−0.53	0.07

Abbreviations: BDD_FVIII_ = B‐domain‐deleted FVIII; FL_FVIII_ = full‐length FVIII; Ig: immunoglobulin; MFI = mean fluorescence intensity; NBA: Nijmegen‐Bethesda assay.

Spearman correlation [*r*_s_] between MFI signal of FLFVIII, BDDFVIII bead and the parameters: FVIII activity, NBA and haemoglobin.

The *p* value and *r*_s_ are highlighted if *p* < 0.05.

The strongest correlation between antibody signal and haemoglobin levels was observed for IgA (BDD_FVIII_ *r*_s_ = −0.53; *p* = 0.07), followed by IgG_2_ (FL_FVIII_ *r*_s_ = −0.39; *p* < 0.001; BDD_FVIII_ *r*_s_ = −0.37; *p* < 0.001). When correlating the FVIII:C with the MFI signal intensity, the analysis revealed a high correlation of IgG anti‐FVIII antibodies (FL_FVIII_ *r*_s_ = −0.45; *p* < 0.05; BDD_FVIII_ *r*_s_ = −0.36; *p* < 0.05) (Table 3).

### 3.4 Domain Specificity of Anti‐FVIII Antibodies

**FIGURE 2:**
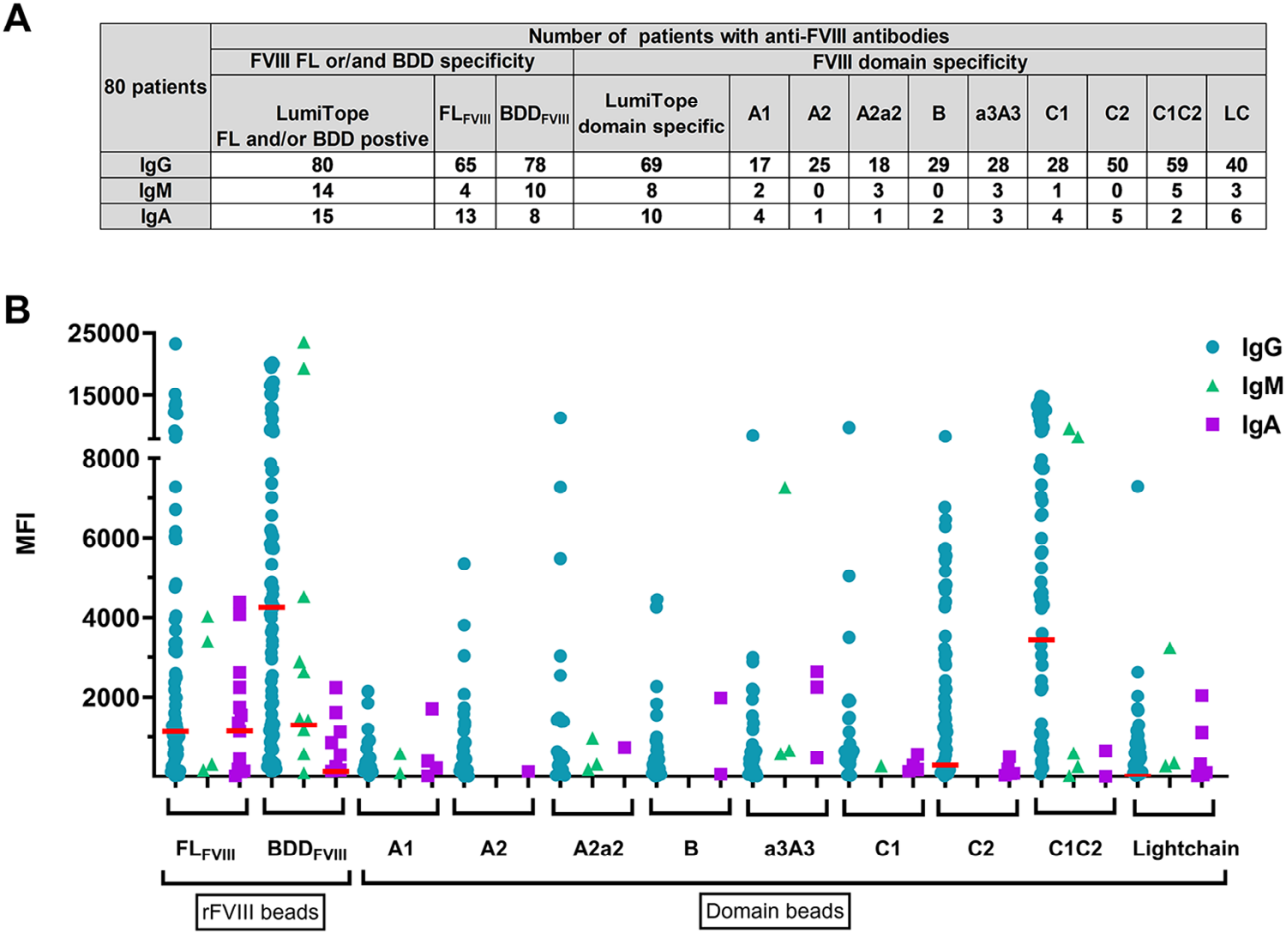
Isotype and Domain pattern of AHA patients. (A) Frequency of anti‐FVIII‐ and anti‐FVIII‐domain specific antibodies in acquired hemophilia A patients. Isotype specific domain profiling was done for 80 patients. The absolute number of patients with antibodies against the corresponding domain is shown. (B) Positive patients with their Ig specific domain pattern and signal intensity [IgG 80 patients, IgA 15 patients, IgM 14 patients]. MFI value of each bead was subtracted by the cut off, negative values were set as zero. Red Line = Median of MFI signal of positive Ig patients; No: number, pos = positive, spec.: specific, LC: Lightchain, IgG, IgM, IgA: Immunoglobulin G, M and A, FL_FVIII_: Full‐length FVIII, BDD_FVIII_: B‐domain‐deleted FVIII, MFI: Mean fluorescence Intensity, anti‐hu: anti‐human, rFVIII: recombinant FVIII products.

When differentiating between antibodies that bind to FL_FVIII_ and BDD_FVIII_ beads, anti‐FVIII IgG and IgM antibodies demonstrated a higher binding for the BDD_FVIII_ beads. In contrast, anti‐FVIII IgA antibodies showed higher positivity towards the FL_FVIII_ bead (Figure 2).

Anti‐FVIII IgG antibodies most frequently targeted the light chain of FVIII, particularly the C1C2 domain (59/80 patients) and the C2 domain (50/80) (Figure 2). Among the 14 patients positive for anti‐FVIII IgM antibodies, 8 exhibited domain‐specific antibodies, with the highest binding to the C1C2 domain (5/8). Furthermore, among the 15 IgA anti‐FVIII antibody‐positive patients, the LC domain was the main target in 6 patients, and 5 patients showed no positive domain binding (Figure 2).

Notably, for 11 patients, no IgG domain specificity was assigned. These patients had a significantly lower NBA median of 4.5 BU/mL compared to the domain‐positive group with 18 BU/mL (data not shown). Of these patients, only one also showed another isotype (IgA) with a specific domain pattern.

Next, Spearman correlation analysis was conducted for anti‐FVIII IgG antibodies between the MFI values of domain and the FL_FVIII_ and BDD_FVIII_ beads. The analysis revealed a strong correlation between the C1C2 (*r*_s_ = 0.48; *p* < 0.001) and the A2 domain (*r*_s_ = 0.47; *p* < 0.001) with the FL_FVIII_ bead. For the BDD_FVIII_ bead, the strongest correlations were found with the C2 (*r*_s_ = 0.65; *p* < 0.001), C1C2 (*r*_s_ = 0.81; *p* < 0.001), and LC (*r*_s_ = 0.53; *p* < 0.001) domains. When correlating domain‐specific antibody profiles with NBA levels, a significant correlation was observed with the C1C2 domain anti‐FVIII antibodies (*r*_s_ = 0.51; *p* < 0.001), whereas no correlation to heavy chain domains was identified (Table 4). Due to the low number of IgA and IgM positive patients, no correlation analysis was performed.

**TABLE 4:** Correlation of anti‐FVIII domain and FVIII bead signal, NBA.

Bead region	Correlation with FL_FVIII_ bead [MFI]		Correlation with BDD_FVIII_ bead [MFI]		Correlation with NBA [BU/mL]	
	*r*_s_	*p* value	*r*_s_	*p* value	*r*_s_	*p* value
A1	0.15	0.18	**0.23**	**0.04**	0.12	0.33
A2	**0.47**	**0.00**	**0.26**	**0.02**	0.14	0.26
A2a2	**0.36**	**0.00**	0.15	0.19	0.03	0.82
B	**0.27**	**0.02**	0.13	0.24	0.19	0.12
a3A3	0.19	0.09	0.03	0.77	0.04	0.71
C1	0.19	0.09	0.18	0.11	**0.28**	**0.02**
C2	**0.40**	**0.00**	**0.65**	**0.00**	**0.42**	**0.00**
C1C2	**0.48**	**0.00**	**0.81**	**0.00**	**0.51**	**0.00**
Lightchain	**0.42**	**0.00**	**0.53**	**0.00**	**0.42**	**0.00**

Abbreviations: BU = Bethesda units; MFI = mean fluorescence intensity; NBA: Nijmegen‐Bethesda assay.

Spearman correlation [*r*_s_] between MFI signal of the domain beads and the parameters: FL_FVIII_ bead signal, BDD_FVIII_ bead signal and NBA.

The *p* value and *r*_s_ are highlighted if *p* < 0.05.

When comparing the domain patterns of antibodies in AHA and CHA patients, distinct differences were observed. While the CHA patients show the most antibodies against the A2 domain (76% CHA; 34% AHA), the anti‐FVIII antibodies in AHA patients are primarily directed against the C1C2 domain (AHA 77%; CHA 49%). Furthermore, the prevalence of anti‐FVIII antibodies towards the B and a3A3 domains was higher in AHA patients compared to the CHA cohort (Figure S2).

### 3.5 Patterns of Multi‐Isotype Anti‐FVIII Antibody Responses in AHA Patients

**FIGURE 3:**
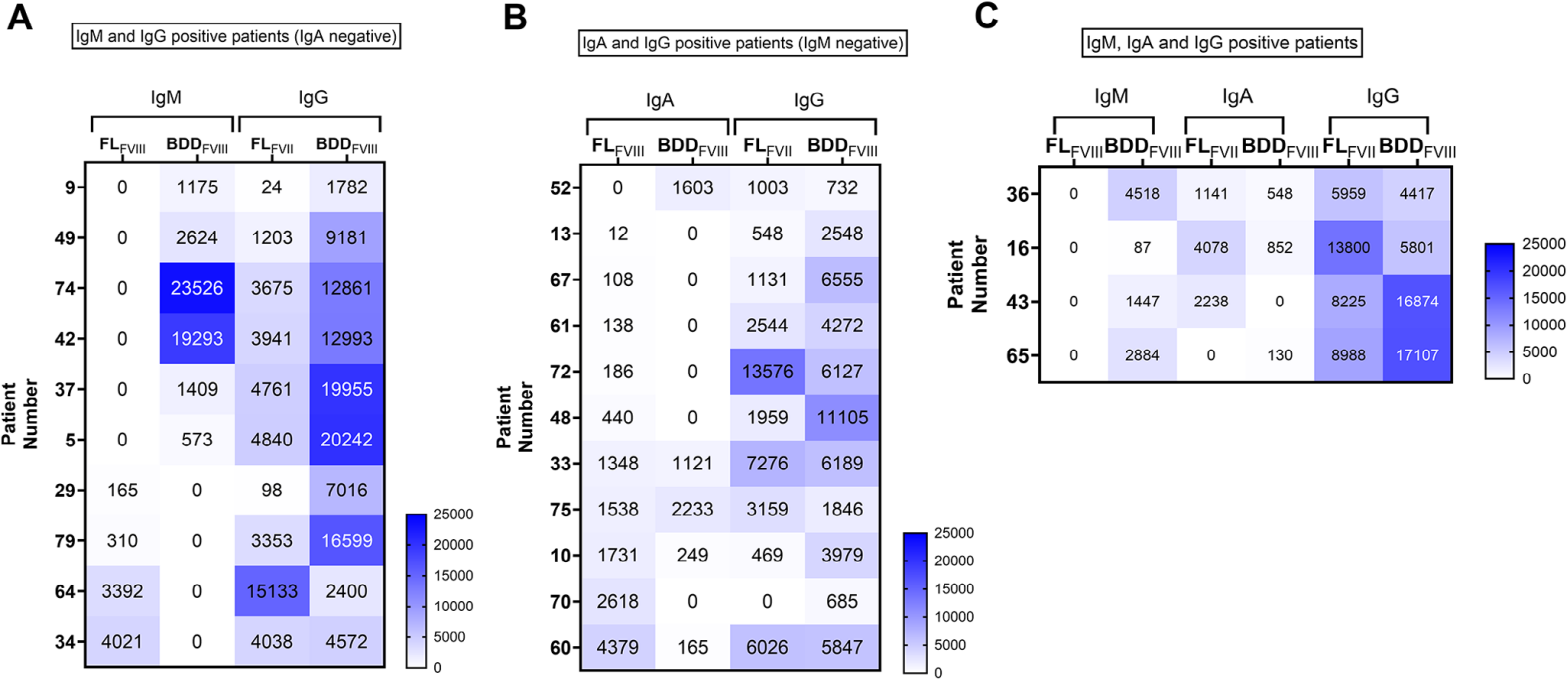
Heat‐Map of patients with multiple positive anti‐FVIII Isotypes (IgG, IgM, IgA). Patients’ MFI values were subtracted from the cut‐off, plotted and colored according to their intensities. The patient identities were anonymized using patient numbers written on the left axis. (A) IgM and IgG positive patients with no IgA anti‐FVIII antibodies. (B) IgA and IgG positive patients with no IgM anti‐FVIII antibodies. (C) IgG, IgM and IgA anti‐FVIII positive patients.

The analysis of patients with multiple positive anti‐FVIII Ig isotypes revealed distinct patterns. Ten patients showed both anti‐FVIII IgM and IgG antibodies. All these patients had positive anti‐FVIII IgG antibodies against both FL_FVIII_ and BDD_FVIII_ beads, whereas only six patients showed a positive result for anti‐FVIII IgM specifically against the BDD_FVIII_ bead. In these patients, the signal strength for anti‐FVIII IgG was also higher on the BDD_FVIII_ bead, mirroring the IgM response. In contrast, the remaining four patients with anti‐FVIII IgM antibodies against only the FL_FVIII_ bead did not show a corresponding trend for IgG antibodies (Figure 3A).

Eleven patients were positive for both anti‐FVIII IgG and IgA antibodies. For these patients, no specific pattern of isotype binding to the FL_FVIII_ and BDD_FVIII_ beads was detected (Figure 3B). Lastly, four patients exhibited positive anti‐FVIII IgG, IgM, and IgA antibodies. In this group, only anti‐FVIII IgM antibodies were detected against the BDD_FVIII_ bead. Two of these patients showed a correspondingly higher signal for anti‐FVIII IgG against the BDD_FVIII_ bead, while anti‐FVIII IgA antibodies demonstrated no consistent association with other isotypes (Figure 3C).

## 4 Discussion

This study provides a comprehensive analysis of the clinical and immunoglobulin characteristics of 80 patients with AHA, highlighting significant correlations between anti‐FVIII antibody profiles and key clinical parameters, including FVIII activity, haemoglobin levels, and NBA titres.

One of the primary findings is that all patients in the cohort were positive for anti‐FVIII IgG antibodies, reinforcing the well‐established dominance of IgG‐mediated autoimmunity in AHA. Notably, subclass analysis revealed a predominance of IgG_4_ (96%) antibodies, consistent with previous reports that IgG_4_ is the most pathogenic isotype in autoimmune conditions [1, 5, 6, 16]. The significant correlation between IgG_4_ and NBA titres (FL_FVIII_ *r*_s_ = 0.45, BDD_FVIII_ *r*_s_ = 0.54) suggests that IgG_4_ antibodies are strongly associated with higher inhibitor levels, confirming their key role in neutralizing FVIII activity. Notably, 19% of healthy individuals develop anti‐FVIII antibodies, though typically not of the IgG_4_ subclass, underscoring the specific role of IgG_4_ in AHA [17]. Although Bonnefoy et al. found a strong link between IgG_2_ and NBA levels in 92% of cases, only 45% of our cohort showed this pattern, with a weaker correlation between the BDD_FVIII_ bead and the NBA titres [5]. Our findings further support that, although anti‐FVIII IgG antibodies correlate with inhibitor levels, they do not directly predict clinical outcomes such as mortality [5, 18, 19].

The six‐month mortality rate (18%) aligns with previous studies reporting 20%–32% within 1 year, emphasizing the need for early diagnosis and effective management [16, 18]. Haemoglobin levels, used as a surrogate for bleeding severity due to variable transfusion practices, proved a reliable clinical marker. The correlation between IgG_2_ antibody signal and haemoglobin (*r*_s_ = 0.39, *p* < 0.001) may suggest a potential influence of this subclass on bleeding severity.

Similar to the GTH‐AH 01/2010 study [16], our results did not confirm a correlation between the IgM titre and the NBA titre shown by Bonnefoy et al. [5] but a similar trend to higher mortality and anti‐FVIII IgM antibodies was observed in our cohort and the QCRI cohort. These differences could be due to variability in isotype binding affinity based on the variability of the immunoassay applied and the NBA method.

On the other hand, Tiede et al., in the GTH‐AH 01/2010 study [16], predicted that IgA‐positive patients had a lower rate of complete remission and mortality. However, in the present study IgA positivity did not correlate with increased mortality, likely due to patient‐specific therapeutic approaches, including treatments for pre‐existing conditions (e.g., cancer, autoimmune therapies) and bleeding‐related interventions (e.g., porcine or human FVIII concentrates, emicizumab). Nonetheless, our data also shows a negative correlation with haemoglobin, suggesting a potential association with increased bleeding risk. Given the limited sample size of IgA‐ or IgM‐positive patients, these findings should be confirmed in larger studies.

Although in this study with the analysis of the sample prior to the initiation of the immunosuppressive therapy, we could exclude treatment‐associated changes of immunoglobulin pattern of anti‐FVIII antibodies. But, due to varied treatment approaches in multimorbid patients, in the frame of this retrospective study, the relationship between treatment success and immunoglobulin patterns could not be evaluated.

Like other studies, we observed variability in FVIII domain targeting, with the C2 and C1C2 domains as primary targets, underscoring their significance in inhibitor binding [7], especially by IgG and IgM antibodies, which bound more readily to the BDD_FVIII_ bead than the FL_FVIII_ bead. This suggests that structural differences between these FVIII forms may influence antibody binding. The strong correlation between C1C2 domain binding and NBA titres indicates that antibodies targeting this domain significantly reduce FVIII:C and may drive disease progression. The question remains why the C1C2 domain is particularly immunogenic. Published research shows that the C2 domain contains immunodominant universal CD4+ epitopes, marked by high solvent exposure and flexible peptide backbones [20]. Additionally, the C1 and C2 domains have exposed surface loops with positively charged residues, facilitating FVIII uptake by dendritic cells [21, 22, 23]. This uptake is influenced by the interaction with von Willebrand factor (VWF), which acts as a carrier protein and prevents FVIII absorption [24, 25, 26].

One interesting finding of the present study was the distinct differences between the anti‐FVIII antibody Ig profile and domain specificity among AHA and CHA patients. While in CHA, anti‐FVIII antibodies primarily target the A2 and C2 domains, in AHA, antibodies predominantly bind to the C1C2 domain. Although both AHA and CHA commonly involve IgG_1_ and IgG_4_, AHA uniquely shows a higher prevalence of polyisotypic responses (IgM and IgA), which are less frequent in CHA.

In summary, our study shows that anti‐FVIII antibody profiles, particularly IgG subclass and domain specificity, are closely linked to inhibitor levels in AHA. However, these immunological markers do not directly correlate with clinical outcomes. There are, however, potential associations, such as IgM antibodies with increased mortality. Future studies should investigate additional factors influencing patient prognosis, including genetic predispositions, treatment regimens, and early immunosuppressive therapy. Understanding these complex interactions will be essential to optimize AHA management and improve patient outcomes.

## Author Contributions

B.P. designed the study. A.C.B. performed the experiments. A.C.B. and B.P. analysed and interpreted data and wrote the manuscript. All authors contributed to data analysis, laboratory/correlative analyses, and manuscript editing and evaluation and read and approved the final manuscript.

## Ethics Statement

This article complies with all ethical requirements for data review and analysis.

## Conflicts of Interest

A.C.B. reports no conflicts of interest. I.M. and K.H. report no conflicts of interest. N.M. received grants/research supports/honoraria/consultation fees from: Bayer, Chugai, CSL Behring, LFB, NovoNordisk, Octapharma, Pfizer, Roche, Takeda and Sobi. T.A. reports having received grants for a patient support association from Bayer, Biotest, Chugai, CSL‐Behring, Grifols, Novo Nordisk, Octapharma, Roche, Swedish Orphan Biovitrum and Takeda, as well as personal fees for advisory board meetings, consulting and/or travel support from Bayer, Biomarin, Biotest, CSL‐Behring, Grifols, Novo Nordisk, Octapharma, Pfizer, Swedish Orphan Biovitrum and Takeda. J.O. reports having received grants for studies and research from Bayer, Biotest, CSL‐Behring, Octapharma, Pfizer, SOBI and Takeda, and travel support as well as personal fees for lectures and advisory board meetings from Bayer, Biogen Idec, Biomarin, Biotest, CSL‐Behring, Chugai, Freeline, Grifols, Novo Nordisk, Octapharma, Pfizer, Roche, Sanofi, Sparks, Swedish Orphan Biovitrum and Takeda. B.P. reports having received grants for research from Biotest and Octapharma as well as personal fees for lectures and advisory board meetings from NovoNordisk and Octapharma.

## Supporting information



Supporting Information

## Data Availability

All data referred to in this article are available for review as required.
