# Supplementary material for: Profiling of Anti‐FVIII Antibodies in Acquired Haemophilia A: ‘Insights into Domain Specificity, Isotype Variability, and Clinical Correlations’
Source: Haemophilia. 2025 May 5;31(4):625–33. doi: 10.1111/hae.70056 (PMC12311880; doi:10.1111/hae.70056)
Supplement: Supplementary file 1 — Supporting Information [file HAE-31-625-s001.docx]

**Supplementary Figures:**

**Supplementary Figure 1. Correlation of anti-FVIII isotype signal and NBA.** Correlation between MFI signal of FL_FVIII_ and BDD_FVIII_ bead to NBA, log-log-fit-line of FL_FVIII_ and BDD_FVIII´_to NBA; ND = not detectable; No fit line was included for IgA and IgM due to the low sample number and non- significant spearman correlation [red = no significance, black = significance)] ;FL_FVIII_ = Full length FVIII, BDD_FVIII_= B-domain-deleted FVIII, IgG, IgG_1_, IgG_2_, IgG_3_, IgG_4_, IgM, IgA = Immunoglobulin G, -G_1_, -G_2_, -G_3_, -G_4_, M and A


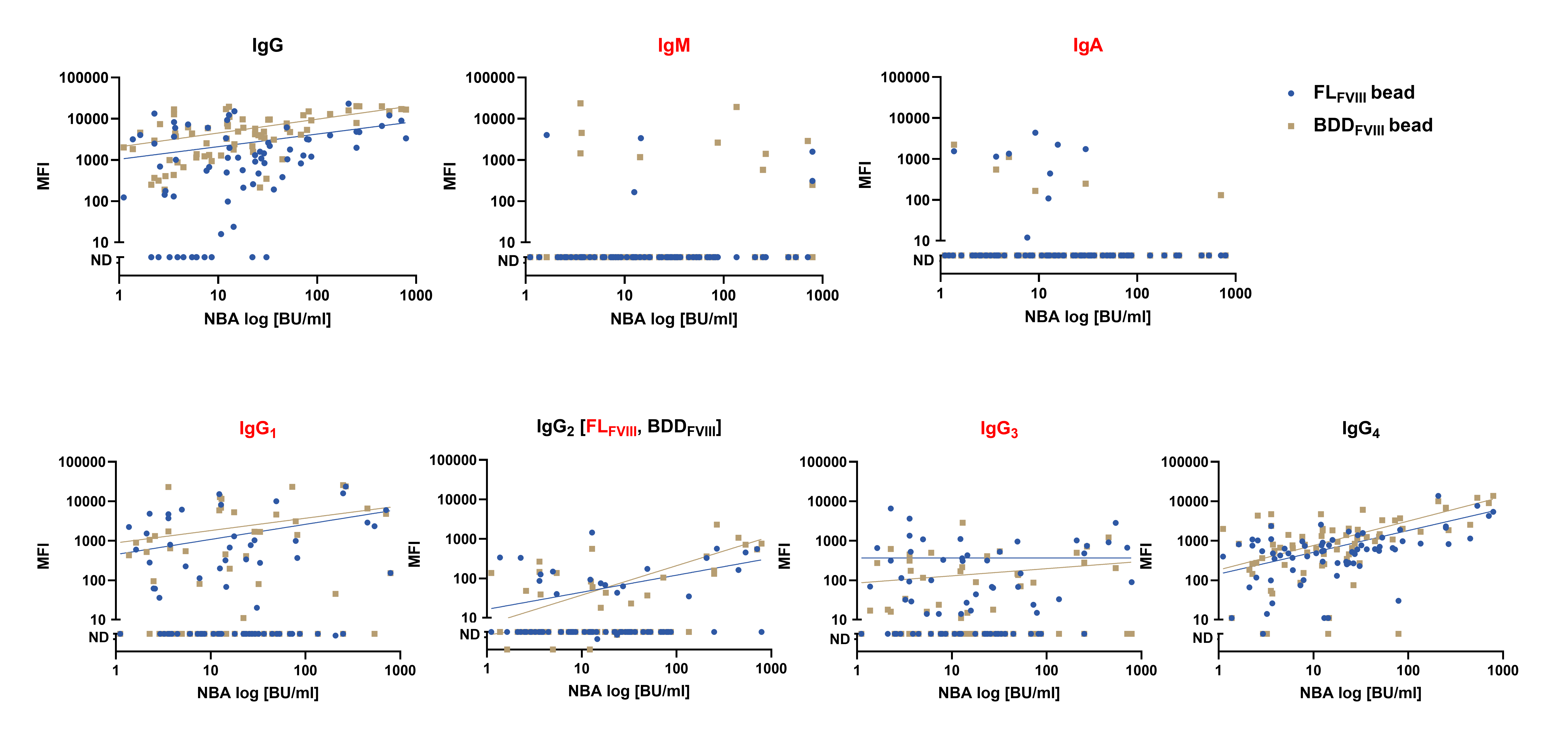


**Supplementary Figure 2: Comparison of anti-FVIII IgG domain signal in AHA and CHA patients.** A) Evaluation of the domain specificity of anti- FVIII antibodies using LumiTope assay. IgG positive samples were assessed for their domain-specific pattern of anti-FVIII antibodies. The results were converted to percentage based on the total number of LumiTope positive samples. B) Median with 95% confidence interval of signal intensity in AHA and CHA IgG anti-FVIII positive patients: MFI were subtracted by the Cut off, negative values were set as zero. FL_FVIII_ = Full length FVIII; BDD_FVIII_= B-domain-deleted FVIII; AHA = Acquired hemophilia A; CHA= Congenital hemophilia A


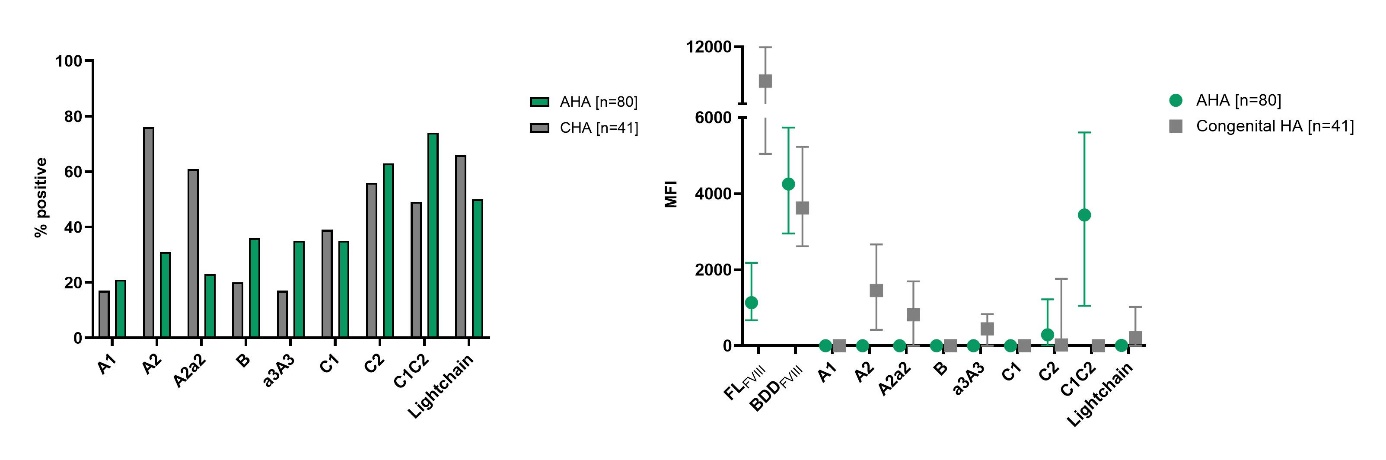


**Supplementary Table 1: Characteristics of the congenital hemophilia A patients.**

| **Congenital hemophilia A patients with inhibitors [n=41]** | | |
| --- | --- | --- |
| Age | Age [years] Median | 16 |
|  | Age Range | 7 months - 81 years |
| Severity | severe [n(%)] | 36 (88) |
|  | moderate [n(%)] | 2 (5) |
|  | mild [n(%)] | 3 (7) |
| Laboratory characteristics | FVIII activity [IU/dl] Median | < 1 |
|  | NBA [BU/ml] Median | 7.14 |

NBA: Nijmegen Bethesda Assay; BU/ml = Bethesda units/milliliter, IU/dl: International units per deciliter
